# Following up

**Published:** 1913-06-14

**Authors:** 


### THE SCHOOL DOCTOR.

### V.—"Following Up."

By "Following Up" is meant the routine procedure used to deal with cases which the medical inspector has certified as requiring treatment for defects found at the examinations. It includes the noting down of such defects on the record cards, the writing out of special follow-up cards, and the reinspection of all cases marked as defective some months after the first examination to find out if these have been treated. In the London County Council area the follow-up cards are yellow, and are kept separately from the record cards, being attached to them only when the case has been treated and no further treatment is required. They have space for special notes with regard to the home conditions, the suggestions for treatment, and for remarks by the doctor or by the Care Committee visitors, to whom the task of following up is entrusted. Where no Care Committee exists the work is largely done by the nurses or by the teachers, but in each case the school doctor has to reinspect the child and to note whether or not adequate treatment has been given. This always demands a re-examination, at which the findings are compared with those of the preliminary inspection; the treatment given is briefly outlined on the card. Some control and care are necessary, and although there exists no well-defined system of treatment at present it is only by following up cases that future improvements can be suggested.

In order, therefore, to be efficient, following up should be looked upon as a very essential part of the routine work of the school doctor. In some areas this is not always the case, and in consequence treatment is relegated to the background while too much is made of the statistics of defects found. Following up has this advantage, that it enables the Care Committees and the school doctor to become acquainted with the home conditions of the child. In some cases in which no treatment is given it is often very difficult to judge whether or not the case is one which can be proceeded with under the Children Act. Where a child's health is obviously suffering from the delay in obtaining treatment for a remediable defect, it becomes a question how far the parents are responsible for such suffering. If they cannot afford to get treatment, either by a private practitioner or by a hospital, the investigation of their means and home circumstances gives the school authorities a definite indication of the fact that they are unable to provide treatment. On the other hand, if the visitor reports that the home conditions are good and that the parents have the means to obtain treatment, but neglect the child, it is obviously a case in which stronger measures can and ought to be taken. In present circumstances it is exceedingly difficult to obtain convictions under the special clause of the Children Act, especially in cases of defective eyesight, and perhaps aural cases, and authorities are naturally chary of instituting a prosecution against parents under these conditions. Doubtless, as the usefulness of treatment for defects discovered becomes more generally acknowledged by the public there will, in course of time, be a change for the better in regard to such cases, but at present we have not progressed so far.

With the actual visiting the doctor has nothing to do; it is in the hands of the nurse and the Care Committee. His functions are to report periodically on the condition of the child found defective at the first inspection. Obviously, the most important part of his work will, therefore, consist in testing the value of the treatment which has been given in particular cases. This impresses upon him the value of keeping fully abreast of the newer methods of treatment and the advances in the various departments of medicine. He has to test the adequacy of the treatment given, and, unless he is familiar with the methods used and knows their limitations and extent, he will fail in properly judging how far treatment is good or bad. In his own mind he must carry some definite standard of efficiency as regards treatment, and this must be his guide in reporting on all cases. Naturally this standard will vary according to the particular case which he has to report upon. Thus, in the case of a poor child with carious teeth, he may find on re-examination that several teeth have been extracted and that a few carious ones remain, which do not need extraction, but stopping. In this instance the parents have done all that they could afford to do, and the case might be noted as "treatment complete and satisfactory." In another child, noted as having adenoids "causing defective nasal breathing and requiring operation," the nasal breathing may have been considerably improved by exercises or other hygienic measures, so that operation is no longer required; this, too, might be classed as a case in which no further treatment is required. In a third, a case of defective vision, the child may have been provided with spectacles; it then becomes necessary to find out whether or not the glasses are correct. If they are, the case is written off as satisfactory; if they are not, the degree of error must be estimated and a note made to the effect that new glasses are required. Such cases in which the treatment given has been incomplete and inadequate are most difficult and unsatisfactory to deal with, since the parents lose heart and grow negligent. Unfortunately, they are relatively common. Dr. Kerr, in his last report on the results of treatment of London school children, gave some interesting particulars of the inadequacy of the treatment given in cases of adenoids and enlarged tonsils. In two cases of a series investigated by Dr. Parsons, the children had been operated upon five times for adenoids, and yet were not relieved, "as their noses were blocked by enormously hypertrophied turbinal bones." It must not be assumed that these results come from treatment received at the hands of general practitioners; as a matter of fact the general practitioner, in Dr. Kerr's statistics, compares very favourably with the large general hospitals in regard to operations for adenoids.

The fullest possible details should be elicited in every case and noted on the cards. Where this is done systematically and with care valuable statistical evidence for future use will be obtained on which the efficiency of the present system may be calculated. We have often urged that the proper way to deal with defective school children is by providing properly staffed school clinics independent of outside influences. Pending the provision of such clinics the only way of improving the condition of the children found defective is for the school doctor to point out the cases where the treatment has not been adequate and to call attention to the manner in which the system fails. This is the value of the procedure of following up, and every school doctor should make it his or her business to deal with it as effectively as possible.

